# Age‐Related Long‐Term Longitudinal Changes in Left Ventricular Mechanical Function in Breast Cancer Patients Undergoing Anthracycline and Trastuzumab Treatment

**DOI:** 10.1002/ccr3.70451

**Published:** 2025-04-18

**Authors:** Begimai Akbalaeva, Priti Singh, Salman Khan

**Affiliations:** ^1^ Department of Clinical Disciplines 1, International Medical Faculty Osh State University Osh Kyrgyzstan; ^2^ Department of Premedical Sciences, and Nutritional, Biochemical and Molecular Sciences American University School of Medicine Aruba Oranjestad Aruba; ^3^ Department of Pathological Processes and Therapeutics American University School of Medicine Aruba Oranjestad Aruba; ^4^ International Medical Faculty Osh State University Osh Kyrgyzstan

**Keywords:** anthracycline, breast cancer, cardiotoxicity, chemotherapy, speckle‐tracking echocardiography, trastuzumab

## Abstract

Age significantly influences the severity of chemotherapy‐induced cardiotoxicity (cardiotoxicity) in HER2‐positive breast cancer patients. Comprehensive cardiac monitoring before, during, and after anthracycline and trastuzumab regimens is essential. Tailored assessments can help identify vulnerable patients, optimize treatment, and mitigate risks of heart complications while maintaining therapy efficacy.

## 1 Introduction

Breast cancer (BC) was the second most commonly diagnosed cancer worldwide, with approximately 1.67 million new cases reported [1]. Anthracyclines (AC) are effective chemotherapy agents with a broad spectrum of action, actively used in the treatment of diseases such as BC. For patients diagnosed with the aggressive HER2‐positive form of BC, the use of AC, particularly in conjunction with trastuzumab (TTZ), markedly enhances both disease‐free survival and overall survival rates [2]. However, their use is limited due to cumulative, dose‐dependent, progressively increasing damage to the heart muscle, which can ultimately lead to heart failure [3]. AC induces oxidative stress in the cardiac muscle, leading to cell damage and potentially causing apoptosis (programmed cell death). This damage is cumulative and can worsen with each subsequent dose of the medication [4]. Treatment with a monoclonal antibody has significantly improved the survival of patients with HER2‐positive BC; however, its association with the risk of developing heart failure and deterioration of left ventricular function raises concerns.

AC with TTZ induced left ventricular dysfunction can be partially or completely reversed if heart failure treatment is started in a timely manner. The cardiotoxic effects can be observed as a decrease in the ejection fraction (EF) of the left ventricle (LV), indicating a compromise in its contraction ability. This condition has the potential to develop into congestive heart failure. In BC, doxorubicin is first‐line drug regardless of the stage of disease. The cardiotoxicity (cardiotoxicity) from AC can be acute and chronic. Researchers performed a randomized random‐effects analysis, suggesting that overweight and obesity are risk factors for cardiotoxicity from AC [5]. Additionally, researchers wrote a systematic review that asserts that diabetes, hypertension, and obesity are associated with an increased risk of AC‐induced cardiotoxicity, which indicates that corresponding protective strategies should be used during and after AC treatment [5]. The mechanism of AC induced cardiac damage has been extensively studied, and it has not yet been fully understood which patients may be affected and at what time during treatment it might occur.

Echocardiography (ECHO) is an essential modality for identifying and treating patients with heart failure. Recent advances in speckle‐tracking echocardiography (STE) have enabled the assessment of layer‐specific myocardial strain, which is a more sensitive marker than ECHO assessing LV systolic function. Global longitudinal strain (GLS) may be more sensitive than LVEF in detecting early cardiac toxicity in oncology patients after chemotherapy and in predicting the risk of heart failure [6, 7]. Assessment of GLS in the time course of AC followed by TTZ therapy to detect cardiotoxicity very early [6]. Normal strain values derived by STE are −19.7% (95% CI, −18.9% to −20.4%) for GLS, −23.3% (95% CI, −22.1% to −24.6%) for global circumferential strain, and 47.3% (95% CI, 43.6% to 51%) for global radial strain [8]. Early detection and monitoring of cardiotoxic effects are vital for mitigating long‐term cardiac sequelae.

In this article, we aim to identify GLS changes with STE in several patients HER2‐positive form of BC before and after chemotherapy, to highlight the importance of an individualized approach and early intervention at the first signs of cardiotoxicity, up to treatment strategies and recovery in cases of serious cardiac complications. This report also addresses the need for standardized monitoring protocols and individualized management strategies, particularly for older patients with pre‐existing cardiovascular risk factors.

## 2 Case Presentation

We presented a 29‐year‐old and a 63‐year‐old female patient with HER2‐positive form of BC. In accordance with the approved treatment based on doxorubicin followed by TTZ per protocol and clinical symptoms, patients have been prescribed adjuvant chemotherapy. Prior to this stage, a detailed assessment of the cardiovascular system was conducted to identify and exclude any potential pathological changes. We wish to highlight that patients did not present with any comorbidities prior to treatment, including hypertension, coronary artery disease, diabetes mellitus, or rhythm disturbances. The younger patient received a cumulative doxorubicin dose of 240 mg/m^2^, administered in four cycles every 21 days, followed by trastuzumab monotherapy. The older patient received a cumulative doxorubicin dose of 300 mg/m^2^, administered in four cycles every 21 days, followed by a combination of doxorubicin and trastuzumab for four cycles, and finally trastuzumab monotherapy. Dose adjustments were made based on body surface area and renal function, with no hepatic impairment noted in either patient.

## 3 Methods (Investigations and Differential Diagnosis)

In Table 1, we presented the clinical analysis outcomes for both patients, before and after 6 months of discontinuing chemotherapy courses. These findings align with the European Society of Cardiology's recommendations for cardiotoxicity monitoring and aid in the baseline cardiovascular risk stratification of HER2‐positive patients [9]. After 6 months of treatment, the young patient lost a little weight, exhibited a slight positive troponin I reaction, and experienced a reduction in serum iron concentration. Upon analyzing, there are no significant changes in the young patient before chemotherapy and after all treatment courses over a 6‐month period. Both patients were examined preoperatively using a Philip Affinity 70 ultrasound diagnostic apparatus equipped with an S5‐1 adult cardiac probe (frequency 2.5–5.0 MHz). The ECHO data of the young woman demonstrate normal values for EF and global strain before and after, although a tendency for reduction can be noted afterwards.

**TABLE 1:** Clinical analysis outcomes for both patients before and after 6 months of discontinuing CTx courses.

Variable	Before chemotherapy		After 6 months of discontinuing chemotherapy courses	
	29‐Year‐old woman	63‐Year‐old woman	29‐Year‐old woman	63‐Year‐old woman
Body mass index, kg/m^2^	28.6	31.4	24.1	32.5
Hemoglobin, g/dL	124	130	112	127
Creatinine, mg/L	59.3	63.4	65.2	65.0
Troponin‐I, ng/mL	< 0.1	< 0.1	0.28	< 0.1
NT‐pro BNP, pg/mL	110.7	426.3	141	15,257.5
Cholesterol	3.2	3.6	3.8	3.8
HLD cholesterol	1.49	1.04	1.14	1.78
LDL cholesterol	3.01	2.90	2.28	2.02
Triglycerides	1.16	2.44	1.2	1.24
Serum iron, μmol/L	12.5	8.5	8.5	10.3
Mg^+2^	1.02	0.99	0.91	0.98
K^+^	4.4	4.3	3.7	4.5
CRP, mg/L	< 5.0	6.1	< 5.0	18.4
hs‐CRP, mg/L	< 0.5	> 5.0	< 0.5	> 5.0
Heart rate, beat/min	69	83	77	126
LVEF, %	69	58	63	27.2
EDV, mL	50	83.1	52	123
ESV, mL	20	40.7	23	89.6
AP2—GLS	−19.6	−18.6	−18.9	−4.7
AP3—GLS	−20.7	−19.3	−14.5	−5.7
AP4—GLS	−21.1	−19.7	−19.6	−4.7
GLS	−20.4	−19.2	−18.2	−5.0
Diastolic dysfunction	−	+	−	+

**FIGURE 1:**
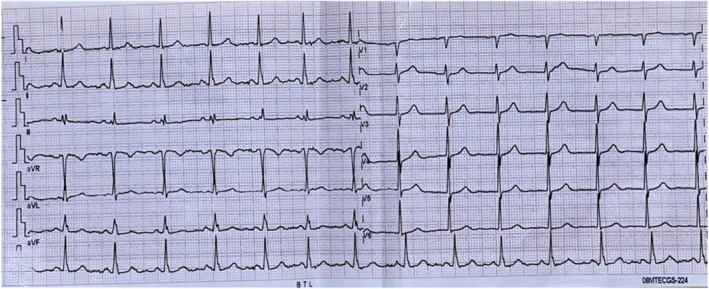
Electrocardiogram (ECG) of a 63‐year‐old female before chemotherapy. The ECG shows sinus rhythm with a heart rate of 83 beats per minute and an incomplete right bundle branch block.

Second patient, a 63‐year‐old woman, presented to the Cardiology Department of Clinic Osh State University (Osh, Kyrgyzstan) in February 2024 due to difficulty breathing for 2 weeks. The patient experienced shortness of breath after minimal exercise, with symptoms progressively worsening over time. Additionally, the patient reported episodes of paroxysmal nocturnal dyspnea and orthopnea. Last year, she was diagnosed with HER2‐positive form of BC and underwent adjuvant chemotherapy, which concluded 6 months ago. Initially, the patient underwent four courses of doxorubicin, followed by four courses of a combination of doxorubicin and TTZ. The final courses were of TTZ monotherapy. Treatment intervals were set at 21 days, with drug dosages individually adjusted based on the patient's diagnosis, weight, and height. An elderly woman was evaluated prior to initiating chemotherapy, with findings showing no significant cardiovascular pathology deviations, except for diastolic dysfunction on ECHO. We want to demonstrate the patient's electrocardiogram (ECG) before chemotherapy, as shown in Figure 1. The ECG displayed a sinus rhythm with a heart rate of 83 beats per minute and incomplete right bundle branch block.

## 4 Results

We examined the patient and compared her clinical test results to those before chemotherapy in Table 1. Six months after the commencement of chemotherapy, laboratory tests and ECHO demonstrated significant changes compared to baseline data:

− The level of natriuretic peptide increased from 426.3 to 15,257 pg/mL.
− C‐reactive protein rose from 6.1 to 18.4 mmol/L.
− EF by Simpson's method decreased from 58% to 27%.
− GLS changed from −19.2 to −5.

**FIGURE 2:**
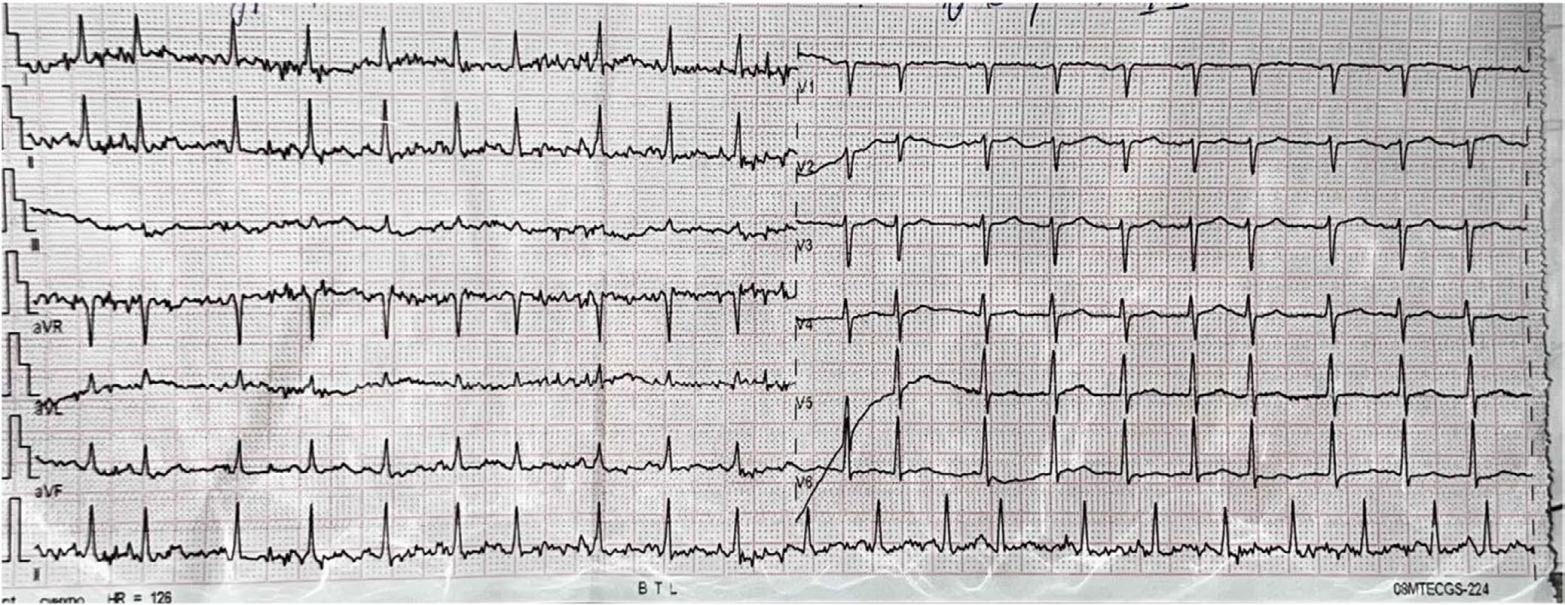
ECG of a 63‐year‐old female upon hospital admission, documenting atrial fibrillation with a heart rate of 126 beats per minute.

Upon admission to the cardiology department of an elderly woman, an ECG was conducted, and we documented atrial fibrillation with a heart rate of 126 beats per minute (Figure 2).

**FIGURE 3:**
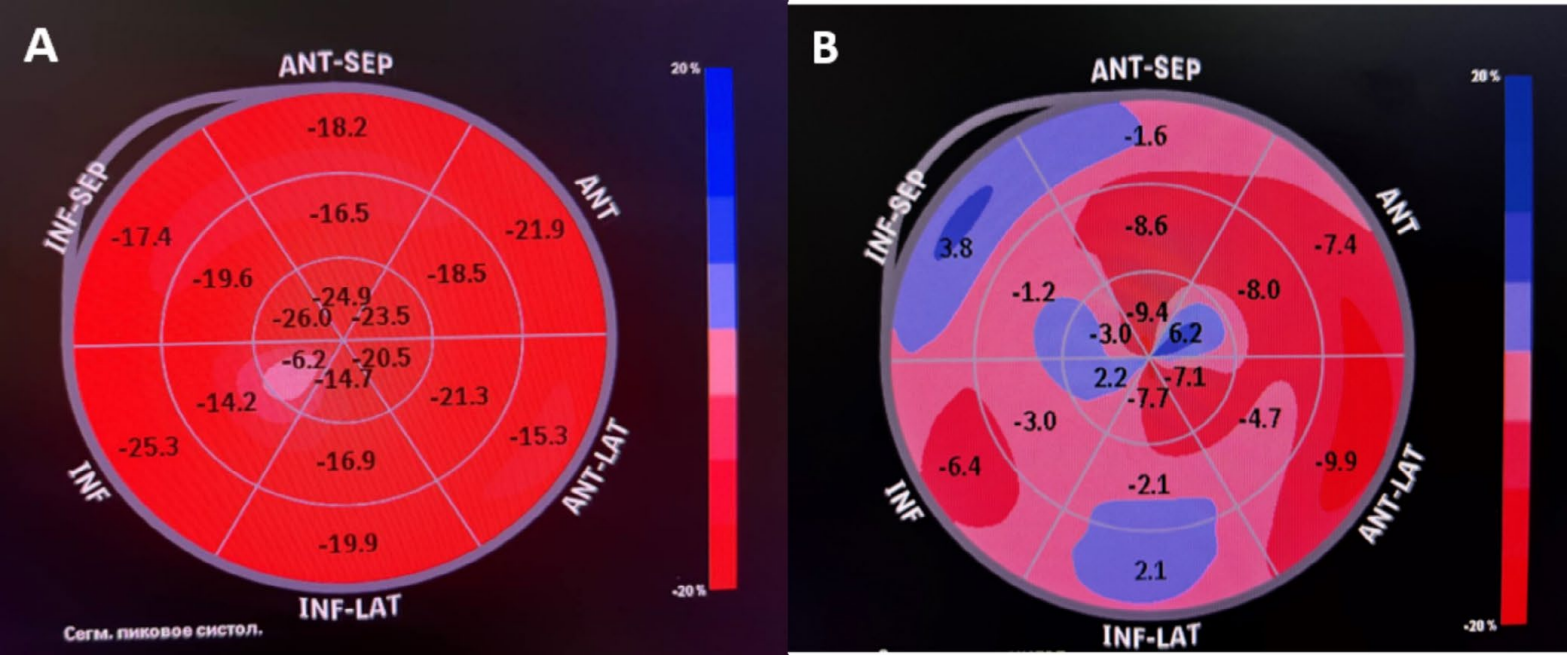
Bullseye plot illustrating the GLS of an elderly woman (A) before chemotherapy and (B) after 6 months of discontinuing chemotherapy.

**FIGURE 4:**
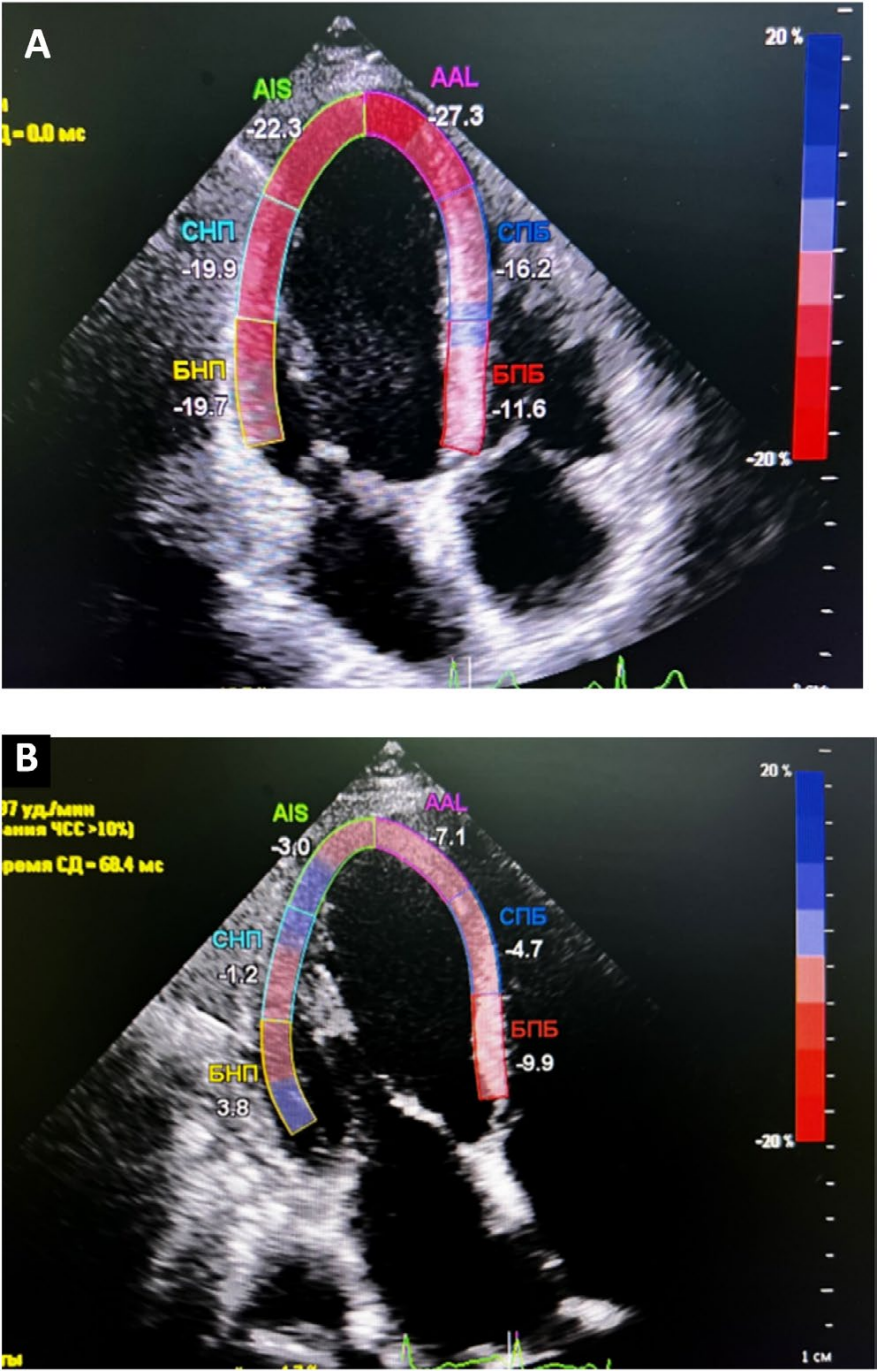
Global longitudinal strain analysis of elderly woman (A) before chemotherapy, (B) after 6 months of discontinuing chemotherapy courses.

The increase in natriuretic peptide level, irregular heart rhythm, and deterioration of ECHO parameters may indicate the development of heart failure, one of the manifestations of cardiotoxicity associated with the use of doxorubicin and TTZ. These drugs are known for their potential to cause cardiotoxic effects, especially when used in combination. The rise in CRP could indicate a systemic inflammatory response, which may also be related to cardiotoxicity or other complications of chemotherapy. The decrease in EF and worsening of GLS reflect the deterioration of left ventricular systolic function, confirming the presence of cardiomyopathy induced by chemotherapeutic agents. In Figure 3A,B shows the change in GLS of an elderly woman is shown before and after 6 months of discontinuing chemotherapy courses. Figure 4A,B shows the strain from an apical 4‐chamber view, assessing the longitudinal deformation of the left ventricle pre‐ and post‐chemotherapy cardiac assessment.

### 4.1 Management

For the heart failure management in older patients includes guideline‐directed medical therapy (GDMT) with beta‐blockers (carvedilol, 6.5 mg), ACE inhibitors (Lisinopril, 20 mg) and mineralocorticoid receptor antagonists (MRAs) (Eplerenone, 50 mg) that were initiated to improve survival and reduce hospitalizations. Diuretics (Torsemide, 10 mg) were initiated to manage congestion, while SGLT2 inhibitors (Empagliflozin, 10 mg) provided additional benefits. Lifestyle modifications, cardiac rehabilitation, and close monitoring for adverse effects were ensured for optimal outcomes in elderly heart failure patients.

## 5 Discussion

This case study underscores the critical importance of vigilant cardiovascular monitoring in patients undergoing chemotherapy for HER2‐positive BC, especially with regimens including doxorubicin and TTZ. In young patients, as demonstrated in the first case, the outcome of chemotherapy was more favorable. The young patient showed resilience to the negative effects of chemotherapy with minimal changes in clinical parameters. This may be due to a higher cardiovascular reserve capacity in young individuals, as well as more active repair and compensatory processes in the body. However, even in this age group, it is important to continue careful cardiac monitoring, as small changes, such as minor weight loss or a weakly positive troponin I reaction, can be harbingers of more serious complications. However, in practice, a clinical case of acute toxic cardiomyopathy was described in a previously healthy young patient who received doxorubicin followed by trastuzumab biosimilar in combination for the treatment of early HER2‐positive BC [10].

The second case, involving a 63‐year‐old woman, underscores the more severe spectrum of chemotherapy‐related cardiotoxicity. The sudden elevation in natriuretic peptide levels, along with longitudinal strain and cardiac rhythm disruptions, indicates the development of chemotherapy‐induced cardiotoxicity as a long‐term complication of chemotherapy. Elderly individuals often have comorbidities and age‐related changes in the cardiovascular system, making them more vulnerable to the negative effects of chemotherapy. In our case, a marked increase in NT‐proBNP levels was observed in the elderly patient (426.3 pg/mL before treatment vs. 15,257.5 pg/mL post‐treatment), suggesting significant cardiac stress. This elevation correlated with a drastic reduction in LVEF (58% to 27.2%) and an increase in end‐systolic volume (40.7 to 89.6 mL), further reinforcing the presence of severe cardiotoxicity. Doxorubicin‐induced cardiotoxicity primarily arises from oxidative stress, mitochondrial dysfunction, and topoisomerase‐IIβ inhibition, leading to DNA damage and cardiomyocyte apoptosis [11]. The addition of trastuzumab exacerbates these effects by disrupting HER2‐mediated cardioprotective signaling, impairing myocardial repair. Older patients experience a more rapid decline in cardiac function due to heightened susceptibility to endothelial dysfunction and mitochondrial injury. Age‐related endothelial dysfunction reduces nitric oxide bioavailability, increasing vascular stiffness and myocardial stress. Additionally, mitochondrial dysfunction in aging cardiomyocytes results in impaired energy production, exacerbating doxorubicin‐induced oxidative damage [12]. These factors collectively accelerate cardiac dysfunction in older patients receiving doxorubicin‐based therapy.

A study by Gąsior et al. involving 155 BC patients found that the left atrial volume index was significantly higher in the group receiving anthracyclines and trastuzumab compared to the group treated with anthracyclines, with or without trastuzumab, and left‐sided radiotherapy [13]. We would like to note that in our second case, the elderly patient initially had left ventricular diastolic dysfunction, which may be an additional risk factor for cardiac damage. Diastolic dysfunction has been increasingly recognized as a predictor of cardiotoxicity in patients undergoing anthracycline‐ and trastuzumab‐based therapy. According to the study by Sawaya et al. [14], patients with baseline diastolic dysfunction are at higher risk of developing heart failure following chemotherapy. In our study, diastolic dysfunction was present in the elderly patient before treatment and persisted after chemotherapy, highlighting its potential as an early marker of cardiotoxicity.

Additionally, echocardiographic markers such as GLS showed significant deterioration in the elderly patient, with GLS values declining from −19.2% before chemotherapy to −5.0% post‐treatment, alongside worsening segmental strain parameters (AP2‐GLS, AP3‐GLS, and AP4‐GLS). This is consistent with previous studies indicating that changes in GLS precede reductions in LVEF and can serve as an early indicator of cardiotoxicity. Similarly, research by Calle et al. reported that combined anthracyclines and trastuzumab therapy resulted in a high cardiotoxicity rate, affecting 20% of patients [15]. In a longitudinal cohort study by Upshaw et al. that included 362 BC patients, abnormal diastolic function was observed in 80% of those treated with doxorubicin or doxorubicin followed by trastuzumab over a 3‐year follow‐up period [16]. Therefore, it is recommended to assess the cardiovascular status of elderly patients before chemotherapy and carefully monitor them during and after chemotherapy.

Our findings support the integration of comprehensive cardiac monitoring, including diastolic function assessment, biomarker evaluation, and serial echocardiography, in patients undergoing anthracycline and trastuzumab therapy. The observed deterioration in LVEF, NT‐proBNP elevation, worsening GLS, and persistent diastolic dysfunction in our elderly patient highlights the multifactorial nature of chemotherapy‐induced cardiotoxicity. Early identification of cardiotoxicity risk factors, especially diastolic dysfunction, can improve patient outcomes by enabling timely intervention and cardioprotective strategies.

## 6 Conclusions

The described cases highlight the imperative for comprehensive cardiovascular evaluation before, during, and after chemotherapy in patients with BC, especially those treated with known cardiotoxic agents like doxorubicin and TTZ. Age plays a significant role in the manifestation and severity of chemotherapy‐induced cardiotoxicity. While younger patients may exhibit resilience to certain adverse effects, older individuals are at heightened risk due to age‐related physiological changes and comorbidities. Thus, these two cases emphasize the need for an individualized approach to assessing and managing cardiotoxicity based on the patient's age. This underscores the importance of considering age‐related aspects when developing strategies for treating BC to optimize outcomes and reduce the risk of cardiac complications.

### 6.1 Limitations of the Study

- *Imaging*: The cardiac MRI is the gold standard; it was not available due to resource limitations. We have emphasized the use of STE as a sensitive alternative for detecting early changes in myocardial function.
- *Follow‐up*: We have acknowledged the limitation of only providing 6‐month follow‐up data and have emphasized the need for shorter follow‐up intervals to assess recovery, progression, or worsening of cardiotoxicity. Future studies should incorporate more frequent follow‐up to better understand the long‐term outcomes.

## Author Contributions

**Begimai Akbalaeva:** investigation, methodology, resources. **Priti Singh:** data curation, formal analysis, investigation, validation, writing – original draft. **Salman Khan:** formal analysis, project administration, supervision, writing – review and editing.

## Ethics Statement

The University Ethical Committee at the International Medical Faculty, Osh State University approved the study (No. IMFRC/case report/18/2025).

## Consent

Written informed consent was obtained from the patients involved in this case report. The patients provided consent for the publication of their anonymized clinical data and associated findings. Efforts have been made to ensure confidentiality, and no identifying information has been disclosed in the manuscript.

## Conflicts of Interest

The authors declare no conflicts of interest.

## Data Availability

The authors have nothing to report.
